# Supplementary material for: MetaRibo-Seq measures translation in microbiomes
Source: Nat Commun. 2020 Jun 29;11:3268. doi: 10.1038/s41467-020-17081-z (PMC7324362; doi:10.1038/s41467-020-17081-z)
Supplement: Supplementary file 10 — Supplementary Data 7 [file 41467_2020_17081_MOESM10_ESM.zip › File2/Confidence_High_Taxonomy/381365_out.krona.html]

Javascript must be enabled to view this page.

members
magnitude
magnitudeUnassigned
count
unassigned
taxon
rank

381365\_out

3

3
2
superkingdom

3
1239
phylum

186801
class
3

186802
order
3

186806
family
2

genus
1730
2

2
1262890

SRS022524\_contig\_number\_7289SRS063040\_contig\_number\_31523
species

1
31979
family

genus
1485
1

1
1262848

SRS144362\_contig\_number\_2407
species
